# Young Londoners, sustainability and everyday politics: the framing of environmental issues in a global city

**DOI:** 10.1186/s42055-020-00036-z

**Published:** 2020-08-28

**Authors:** James Sloam

**Affiliations:** Department of Politics and International Relations, Royal Holloway University, Egham, Surrey TW20 9BW UK

**Keywords:** Young people, Sustainability., Politics., Environment., London., Global Cities.

## Abstract

**Background:**

Young people have become disillusioned with mainstream electoral politics in established democracies, and are increasingly likely to engage in democracy on a case-by-case basis in *issues* that hold meaning for their everyday lives. These issues are most likely to be local and involve interconnected economic, social and environmental concerns.

**Results:**

The following study contributes to the understanding of the everyday politics of young people within a single urban environment (London) regarding sustainability-related issues. It departs from many previous studies of sustainability by employing a youth-centred approach, focussing on the *authentic* voices of young Londoners. The article shows how narrative structures can provide an effective way of understanding everyday politics and the interconnectedness of key issues through the words of these young people.

**Conclusions:**

The analysis identifies young people’s relatively low prioritisation of most environmental issues – due to an overwhelming sense of economic precarity and a lack of opportunity to reflect upon the relevance of environmental issues for their everyday lives. The article emphasizes the importance of opening up spaces for deliberation and channels for youth engagement, to embed sustainability in global cities.

## Plain English summary

Young people have become increasingly disillusioned with politics and political parties over a number of decades. They are now much more like to be interested in issues than join political parties, and are interested in a wide range of subjects – from housing, to crime, to the environment.

This article looks at how young Londoners understand the role of environmental issues within the context of their everyday lives – the interconnection between issues such as housing and crime and perceived threats to the natural environment.

The article finds that, whilst young people care about environmental issues, they tend to be consumed by day-to-day problems of *just getting by*. Nevertheless, young Londoners are quick to express their views and get involved in environmental questions, if they are given the opportunities to discuss them with their peers.

## Introduction

Young people have become increasingly disconnected from mainstream electoral politics in established democracies. Over the past four decades, we have witnessed a transition from formal political participation (voting and party membership) to issue-based participation through campaigns and social movements and citizen-to-citizen interactions [18]. Today’s young people tend to engage in democracy on a case-by-case basis in issues that hold meaning for their everyday lives [1]. Their politics is shaped by ‘everyday experience’: these ‘everyday makers are project-oriented and want to deal with common concerns concretely and personally rather than abstractly and ideologically … [and] they do not want to spend their precious time participating in formal political institutions’ ([5]: 162, 167).^1^ Those common concerns are most relevant in local areas – the cities, towns, neighbourhoods where citizens reside [15].

This study contributes to the understanding the everyday politics of young people (15 to 24 year-olds) within a single urban environment (London) regarding sustainability-related issues. The United Nations Sustainable Development Goals (SDGs) set out a broad range of economic, social, political and environmental issues, which can be examined. But the SDGs inevitably suffer from measurement problems [27]. Individual goals are often measured separately (driven by so-called “objective” quantitative indicators), so that the interdependence between these problems and the *authentic* voices of young people are often lost. The following investigation seeks to overcome these problems by focussing on *youth voice*, and sets out to address two key questions:

1. How do young Londoners understand the role of environmental issues within their everyday lives?
2. How are environmental issues framed within broader narratives of everyday politics?

This article shows how narrative structures can provide an effective way of understanding everyday politics and the interconnectedness of key issues through the voices of young Londoners. It examines how they frame environmental issues within the context of other existential (economic and social) challenges they face in an expensive, fast-moving and often disconcerting global city. The analysis identifies young people’s relatively low prioritisation of most environmental issues – due to an overwhelming sense of economic precarity and a lack of opportunity to reflect upon the relevance of environmental issues for their everyday lives. The article emphasizes the importance of opening up spaces for deliberation (in schools, universities and other youth institutions) and channels for youth engagement in public policy, to better embed future-oriented, sustainable public policy in global cities.

## Young people in a time of crises

Why study young people? Younger generations have the greatest vested interest in sustainable public policy – in the long-term future of our societies and planet. And, studies of political socialization show that the 15–24 age period is most important in the formation of political attitudes and identities [11, 17]. People are particularly sensitive – economically, socially and politically – to new events and circumstances, and open to new ideas, during their transition into adulthood [2, 12].

Global cities, such as London, have become a prominent feature of post-industrial societies [24]. These places act as hubs for international trade and as beacons for elites and aspirational classes. They are diverse and cosmopolitan, but – at the same time – contain vast inequalities in wealth, power and influence. This is particularly true for young people from deprived backgrounds and traditionally marginalised communities. As these cities continue to prosper, young people are often pushed to the margins by issues that affect their everyday lives – from rising house prices, to the prevalence of low-paid, short-term jobs, to the increasing costs of higher education [16]. This situation has been exacerbated by the financial crisis and austerity. Whilst increased *precarity* is a common feature of most post-industrial democracies [6], the UK has experienced relatively deep cuts in public spending that have had a dramatic impact upon younger generations [22]. For example, cuts to youth services and education budgets have increased the likelihood of social exclusion and related problems such as poor mental health and violent crime. And, the Covid-19 pandemic has made the economic situation much worse for young people in particular – social distancing restrictions have led to large-scale job losses and pay cuts, as well as the reduction of key support services for vulnerable young people in the capital [23, 32].

The worsening economic and social conditions for younger generations after the Great Recession ignited a wave of protest of the ‘outraged young’ – from the Spanish Indignados on the streets of Madrid, to Occupy Wall Street in Manhattan, and protests against the trebling of university tuition fees in London – against economic and political elites [3, 8]. But this wave of protest appears to have had little influence upon the conduct of public policy.

Recent research has identified the increasing prevalence of postmaterialist and cosmopolitan values amongst younger generations [4, 19]. Young people are, on the whole and in most established democracies, more socially liberal and outward-looking (relaxed about immigration and supportive of cultural diversity) than older generations. In political terms, younger generations have mobilised against a rise in authoritarian-populist politics, including strong youth opposition to Brexit (in the 2016 referendum 76% of 18 to 21 year-olds and 82% of full-time students supported UK membership of the European Union) [4]. More recently, many young people protested against government inaction on the *climate crisis*. In 2018 and 2019, young activists across the world joined a ‘schoolstrike4climate’ to shine a light on the issue (inspired by the actions of Greta Thunberg in Sweden) [26]. This green wave was also in evidence in the May 2019 European Parliament elections, when environmental parties across Europe profited from a surge in youth support [29].

At first glance, these economic and environmental crises create competing demands. Whilst some young people (disproportionately students) seem to be more aware of environmental crises and politically active in supporting Green politics, many others (especially young people not in education) are preoccupied by existential economic and social issues – to literally *put a roof over their heads* – that are particularly prominent in a high-cost, fast-moving urban environment such as London. Subsistence costs in global cities are very high and place intense pressure on young people to be successful in their transition to the job market. This article will show how – given the space to reflect upon their concerns and construct narratives with one another – these demands are deeply interconnected.

## Methods

Studying the political views and practices of the most diverse cohort of young people is a complex task [25]. Young people’s understandings of the world around them are multi-layered – from the individual and family, to the community and neighbourhood, to international questions of human rights and environmental justice – and interconnected across clusters of issues. They are highly dependent upon an individual’s economic circumstances, their sense of identity and the challenges they face in a particular geographic location. So, it is important that we, as researchers, become ‘literate’ in young people’s views [21], in the conceptions and narratives regarding their everyday lives, so that we ask the right questions. Much of the literature on the political views and engagement of young people relies heavily on survey data, which negates these complexities and undervalues the importance of space and local context in defining young people’s everyday lives [30].^2^

The ‘Young Londoners – Sustainable Cities’ project, supported by the Greater London Authority (GLA), employed a youth-centred approach to investigate the views of young people within a distinct urban environment. The fieldwork began, in autumn 2018, with 30 in-depth interviews with a diverse group of 15 to 24 year-olds working for the GLA Peer Outreach Team. As there is no in-depth survey data on the issues that young Londoners believe are important, we begun by exploring these issues with young members of the Peer Outreach Team. The interviewees were predominately female, from ethnic minority backgrounds, and relatively civically (but not politically) engaged.^3^

These discussions helped to establish the main themes that would be discussed with their peers in ‘café-style’ focus groups at a two-hour event at City Hall. The participants in the focus groups, brought together by the Greater London Authority, were 15–24 year-olds from community groups and youth organizations. Replicating the demographics of young Londoners, approximately half of the participants were men and women and half came from non-white, ethnic minority backgrounds. The ‘world café’ approach [7] was used to encourage ‘naturalistic and conversational approaches to gathering data’ ([31]: 339) – ‘everyday political talk’ through which the young people could ‘construct their identities [and narratives], achieve mutual understanding … [and] form considered opinions’ ([14]: 51). Each themed discussion was led by a young facilitator (one of the interviewees from the Peer Outreach Team, trained by the author), who moved between ten tables of seven to twelve young participants, exploring the issues and potential solutions through open discussions. The facilitators also used a Lego brick exercise, to stimulate discussion amongst their groups. All of the quotes and reflections discussed in the results section come from this World Café event.^4^

The qualitative data was captured through a narrative framework, complementing the naturalistic discussions encouraged in the café-style focus groups. Adapting the analytical frameworks of [20, 28], I emphasize the importance of “problematization” (how environmental issues were perceived within the broader framework of everyday politics), “emotion” (the articulation of strength of feeling, placing greater emphasis on certain problems) and “power” (the sense of agency or helplessness that was embodied in the narratives). Meanwhile framing refers to the act of ‘*selecting and highlighting some facets of events or issues, and making connections among them so as to promote a particular interpretation, evaluation, and/or solution*’ [10]. Using these features of narratives and framing as an analytical guide, the following section explores how young Londoners frame environmental issues within a global city.

## Results: young Londoners framing of the environment

One of the main findings that emerged from the focus groups was that – whilst young Londoners care about the environment – it is not their most pressing daily concern.^5^ One female participant (aged 19) remarked: “It is a very ignored issue, because in London now there are more serious issues that we focus on which are life-dependent … like housing, or poverty, or crimes”.

Several participants also made the point that that they had a tough time convincing other young people to respect the environment. Thus, many young Londoners had not previously reflected, in any great depth, on environmental issues prior to the City Hall event, and *problematized* the issues involved on the day with their peers (rather than coming to the event with pre-constructed narratives).

Yet environmental concerns were paramount for a significant minority - almost all of them young women. One female focus group participant (aged 17) built her Lego model to depict the problem of ‘plastic’ – a ‘green world’ with ‘everything on top plastic’: ‘the issue is that we have too much of it and we should try to reduce it. Not just by recycling … but to try and stop using plastic’. Another female participant’s (aged 18) Lego model portrayed:

‘a person and their little boat and they’re trying to get away from all the trash and pollution that we’ve created … that’s a little bug floating away … He’s trying to get to the greener space where everything’s very clean and the environment is looked after.’

These comments were symptomatic of a feeling of *powerlessness* and lack of agency, and the emotional state of being overwhelmed by the scale of environmental challenges.

The young Londoners in the focus groups expressed differences regarding attitudes depending on their gender, but social class and geographic location also appeared to be important, placing environmental issues into the broader context of urban life. Poor environmental standards were associated with poor and ignored neighbourhoods that people in power did not care about. Several participants talked about the lack of recycling facilities and loads of rubbish on the streets in ‘shit neighbourhoods’:

‘there are certain places in my community where you have junkies who just leave their needles, leave everything, and there needs to be safer ways for local authorities to clear that within a quick amount of time’ (female, aged 17).

A large majority of young Londoners believed that poverty and lack of public provision were the driving forces behind the challenges they faced. One young woman (aged 19) talked about how money affected access to nature – the fact that the authorities charge to sit down in the chairs in Hyde Park was viewed to be fundamentally unfair on those who are poor or have a disability/health problem: ‘the environment is a big part, because it is constantly adding and decreasing to either our emotional state or our health and the way we function as a society.’

Participants felt that some, poorer neighbourhoods, were bypassed, frozen out and left behind in the growth of London. This connects with the idea of ‘power’: richer people from wealthier boroughs receiving better services from the state. They talked about being ignored and frozen out of public services, and how this impacted upon their everyday lives. For instance, to promote cycling, one young man (aged 18) commented:

‘you’ve got the Boris bikes, which are great, but they’re only for a certain area. It doesn’t come to the hood … They need to bring it to South London – Lewisham, Hackney – where people do actually need this transportation to get into London.’

One young woman (aged 21) complained that:

‘bus stops are closing, buses are passing by in neighbourhoods that are deemed as unsafe or deprived in a way … for example, they’ve closed off the back of Stratford, so you only see the front, the Westfield side’.

Some suggested that 24-h public transport would help alleviate their fears and also alleviate some of the causes of crime.

Young Londoners placed a high value on parks and nature – ‘a space with your community that really de-stresses you’ (female participant, aged 18). This is interesting, because mental health – alongside housing and crime – was one of the top priorities identified in the subsequent survey of young Londoners. However, many young people also thought that this was something London was already very good at: ‘I feel, like in terms of green space, London is doing very well’ (male, aged 24).

Nevertheless, a lack of public provision undermined the space for young Londoners within their urban environment. Discussing the causes of crime, a common response was that young people (especially those without much money) had few places to go and this led to trouble. One young Londoner (male, aged 23) explained how he thought this worked:

‘I think the deeper problem within this is the fact that there is no youth provisions or youth centres. Like my borough, Waltham Forrest, there is not one single youth centre where young people can go to, and they congregate in McDonalds … [which] causes altercations and arguments … It’s a cycle, basically, a vicious cycle.’

He continued:

‘where I used to live, like in Brent, there were loads and loads of youth centres so everyone would go up and we would chill … we had the police in to come and talk to us and it cleared the atmosphere … [after talking about youth centre closures] I feel that if they brought them back they would definitely do some good’.

The café-style focus groups acted as a deliberative forum – young Londoners felt the experience (of constructing narratives) to be empowering – and led to many proposed solutions to environmental problems. Two members of the focus groups raised the idea of boycotting companies with insufficient environmental standards and pressurizing government and the London authorities into not investing in those companies. One young woman (aged 21) expressed her support for ‘Starting new local schemes for growing your own produce within the community … [which can] reduce the amount of waste we produce.’ This received strong approval from other participants in her group. Others supported the idea of community groups needing to encourage young people to ‘take responsibility’ and become involved in the maintenance of parks, communal gardens, and so on. Another suggestion was the building of more dedicated cycle routes to ‘encourage young people to cycle more’ (male, aged 16) as riding ‘on a busy road in the middle of central London is not safe’ (female, aged 17).

Young people’s narratives were strongly associated with the struggle to ‘get by’ – here the difficulty of transitioning into adulthood was a prominent topic – particularly for young people from poorer backgrounds. There were critical junctures in young people’s lives. Young Londoners noted the lack of support in their transitions to adulthood (for those turning 18) across several issues: for example, advice for obtaining housing, benefits and mental healthcare; and, coping with new costs, such as prescription charges, local travel and university tuition fees. A participant (male, aged 21) added that ‘if you’re a young person who hasn’t got any education and are just trying to get by and work a normal job, London has priced you out’. For example, the issue of a “cliff-edge” (on turning 16 and, then, 18) for young people (as they transitioned into adulthood) was raised with regard to public transport: ‘It’s not reasonable and it feels like it’s suddenly changed … from going to school to suddenly going to college and having to spend so much money to get to college.’ (female, aged 18). Thus, transition was a key dimension of young people’s narratives.

## Discussion

The young Londoners who participated in the project provided rich narratives about the challenges they faced in their everyday lives. However, the *problematization* of environmental issues was, on the whole, underdeveloped. Environmental issues were simply less tangible than existential economic and social problems faced during their transition to adulthood (in the context of the financial crisis and austerity in public spending). This must be placed within the context of increasing precarity for younger generations in the UK.

When concerns were raised about environmental issues – over the level of pollution in the city, insufficient and confusing provision of recycling, and the availability of green spaces – the narratives were framed in a deeply personal way. For example, those upset with air pollution complained of its effects on their, or a family member’s, asthma.

The spillover from economic and social issues to environmental issues (more broadly defined) was prominent in the discussions – especially when regarding the local neighbourhood. In this regard, young Londoners constructed narratives about ‘shit neighbourhoods’, and being left out or left behind. Another common feature of the discussions was the impact of green spaces on mental health.

Young people in the focus groups shared a sense of *powerlessness* – a feeling that they lacked the ability to influence big social, economic and environmental issues. Either they did not know who to blame or did not know how to engage. For those who possessed a strong sense of “internal efficacy” (belief in their ability to make a difference), they often became frustrated by insufficient support from public authorities e.g. in setting up community groups. One female participant (aged 18) shared her experience:

‘I got saved from a lot of rubbish by my [youth centre] basketball coach [who] literally supported the whole team through a lot of things’; ‘me and a friend of mine started a network for young women, because there wasn’t any youth clubs and we literally funded it ourselves until we were both broke and could not keep doing it.’

Education and deliberation more generally had a pivotal role to play in lifting the temporal horizons of young people, and allowing them construct narratives regarding environmental and sustainability-related challenges. Indeed, the focus groups themselves acted as deliberative spaces, where the researchers saw – in real time – these narratives develop. The world café-style focus groups, using young facilitators to lead the discussion, were incredibly productive. And, the researchers were surprised (after initially being quite cynical) about the effectiveness of the Lego-building exercise, which stimulated discussion and encouraged contributions from participants who were less confident about articulating their views. This supports the findings of previous work: that ‘young citizens need democratic language, tools and ways of understanding their situation to recreate a common and more sustainable world’ ([13]: 4–5).

In this regard, schools can play a key part in preparing young Londoners for civic life. Indeed, young Londoners themselves recognised that they often lacked the knowledge or the skills to participation: ‘all the politics I know is self-taught … I hated politics … it scared me … didn’t relate to the lingo then … if you really want people to vote and be part of the political system, you have to explain it to them … ’ (female participant, aged 21). Participants felt that schools should provide more knowledge about the environment, importance of recycling, and the effects of pollution.

Youth agency was strongly related to *scale*. Almost all the solutions proposed by young Londoners were local ones. And, young Londoners were most likely to feel a strong sense of efficacy about getting things done on this plane – in their communities and neighbourhoods as the most likely scale upon which problems can be solved. A number of focus group participants stated they felt best able to ‘make a change’ through social work and participation in community projects (e.g. youth work, mentoring). Young Londoners presented various ideas for solving what they saw as a housing crisis: including, ‘better schemes for social tenants to be able to purchase their properties’ (male, aged 24); and, ‘community-led self-build projects’ for affordable homes using recycled materials, such as those led by the Rural Urban Synthesis Society in Lewisham (female, aged 24). However, many did not feel that they had enough time or resources to contribute.

However, the political context for the current situation is not helpful. Older generations have dominated the political fortunes of the UK, supporting Brexit and Conservative-led austerity against the electoral preferences of younger generations. And, the first-past-the-post electoral system restricts the potential growth of the Green Party, which has happened in several other European countries. Furthermore, the centralized system of governance in England and the slashing of local council budgets over the past decade has created a situation whereby the opportunities for meaningful engagement with policy-makers at the local level are few and far between. These factors contribute to low prioritisation of environmental issues in public life and lack of scaffolding to support youth participation in environmental affairs.

## Conclusions

The study finds that it is almost impossible – and counterproductive – to disentangle social, economic and environmental issues. Regarding public policy, interconnected issues require interconnected solutions. The key, of course, is to build bridges between young people and public policy, which can only be achieved by providing effective educational spaces to reflect upon the challenges they face and establishing clear pathways to engagement with local or city-wide authorities. Politicians and officials in London and other large cities need to work harder to channel youth voices into the policy-making process (following examples of good practice from around the globe – [9]) if they are to construct genuinely sustainable public policies.

## Data Availability

The data from the interviews, focus groups and survey has been deposited with the London Sustainable Development Commission and the London Data Store, in accordance with Greater London Authority policy. The Commission has decided that all the data will be publicly available (on request) from the store 6 weeks after the report is released (due 16 September). As lead consultant, the Commission has accepted the author’s right publish the data in academic outlets.

## Footnotes

1 Li and Marsh [15] identified 37% of the British population as everyday makers, rising to 44% for 16 to 24 year-olds (compared to a third of those over 60).

2 Furthermore, these surveys often include only a couple of hundred ‘young adults’ (18 to 24 year-olds). The majority of studies in political science use survey data to investigate the policy priorities of young people. Unfortunately, these studies only use nationally representative samples, with unrefined questions (e.g. ‘how important do you think the environment is as a political issue?’), so are unable to capture the localized and nuanced realities of everyday politics.

3 Each interview lasted approximately 30 min, was conducted by the author or project research assistant, and took place in City Hall. The interviews were recorded, and then transcribed by a professional transcription company.

4 The qualitative work fed into the development of nuanced questions fielded in an *n* = 2002 survey of 16–24 year-olds.

5 The one exception to the low prioritization of environmental issues, confirmed in our subsequent survey of young Londoners, was air pollution (ranking a little below crime, housing and mental health issues as a priority for government).
